# FinO/ProQ-family proteins: an evolutionary perspective

**DOI:** 10.1042/BSR20220313

**Published:** 2023-03-01

**Authors:** Zhen Liao, Alexandre Smirnov

**Affiliations:** 1 UMR7156 - Génétique Moléculaire, Génomique, Microbiologie (GMGM), University of Strasbourg, CNRS, France; 2 University of Strasbourg Institute for Advanced Study (USIAS), France

**Keywords:** evolution, FinO, ProQ, RNA chaperone, RNA-binding proteins

## Abstract

RNA-binding proteins are key actors of post-transcriptional networks. Almost exclusively studied in the light of their interactions with RNA ligands and the associated functional events, they are still poorly understood as evolutionary units. In this review, we discuss the FinO/ProQ family of bacterial RNA chaperones, how they evolve and spread across bacterial populations and what properties and opportunities they provide to their host cells. We reflect on major conserved and divergent themes within the family, trying to understand how the same ancestral RNA-binding fold, augmented with additional structural elements, could yield either highly specialised proteins or, on the contrary, globally acting regulatory hubs with a pervasive impact on gene expression. We also consider dominant convergent evolutionary trends that shaped their RNA chaperone activity and recurrently implicated the FinO/ProQ-like proteins in bacterial DNA metabolism, translation and virulence. Finally, we offer a new perspective in which FinO/ProQ-family regulators emerge as active evolutionary players with both negative and positive roles, significantly impacting the evolutionary modes and trajectories of their bacterial hosts.

## Introduction

Bacterial cells heavily rely on post-transcriptional mechanisms of gene expression regulation that involve diverse RNA-binding proteins (RBPs) and small noncoding RNAs (sRNAs) [1,2]. The rise of genome-wide approaches, such as the complexomic methods Grad-seq and GradR and the phase separation-based OOPS, TRAPP and PTex, enabled the discovery of a large repertoire of bacterial RBPs [3–8]. Among them a special class of globally acting RBPs stands out. Such well-characterised RNA chaperones as Hfq and cold-shock domain proteins, translational repressors of the CsrA/RsmA family, and the key ribonucleases RNase III, RNase E and RNase J, bind and regulate hundreds of transcripts [9–15]. As critical hubs of cellular RNA-protein networks, these proteins pervasively control bacterial physiology and ensure its adaptability to changing environmental conditions.

Due to their central role in the realisation of gene expression programmes, such RBP hubs represent an inherent vulnerability of post-transcriptional networks [16,17], and their loss is typically associated with dire pleiotropic phenotypes. Consequently, they are usually preciously preserved functional units of bacterial cells, subject to strong purifying selection. On the other hand, by critically contributing to the fitness of their hosts, these pervasive regulators likely influence the way bacteria and their transcriptomes evolve to adapt to a wide diversity of ecological niches, including complex symbiotic and pathogenic lifestyles. This makes global RBPs potentially important evolutionary players.

Last years have seen a surge of interest in the FinO/ProQ family of RNA chaperones [18]. This eclectic group of RBPs widespread in Proteobacteria includes both highly specialised plasmid-encoded regulators, such as FinO, FopA and PcnR [4,19,20], and globally acting sRNA- and mRNA-binders, such as ProQ, which make part of the core cellular proteome and post-transcriptionally control multiple regulons [21]. Today, the FinO/ProQ family boasts a good collection of solved structures covering multiple variations around the unique FinO/ProQ domain they all share [22–25,63]. This structural diversity is backed by a wealth of structure–function information at the biochemical, genetic, molecular biology and physiological levels, which shed light on how FinO/ProQ-like proteins deal with their RNA ligands to influence the expression of their target genes in various biological contexts. The large body of available data makes this group of RBPs a particularly attractive model to address so-far unresolved questions about the evolutionary aspects of global post-transcriptional regulators in bacteria.

In this review, we will discuss the place of the FinO/ProQ family in microbial evolution from two standpoints, by considering them as (i) evolutionary targets carved by natural selection to meet the needs of their host cells and (ii) as active players shaping the regulatory repertoire and the evolutionary trajectories of their hosts.

## Biological functions of FinO/ProQ-family proteins

**Figure 1:**
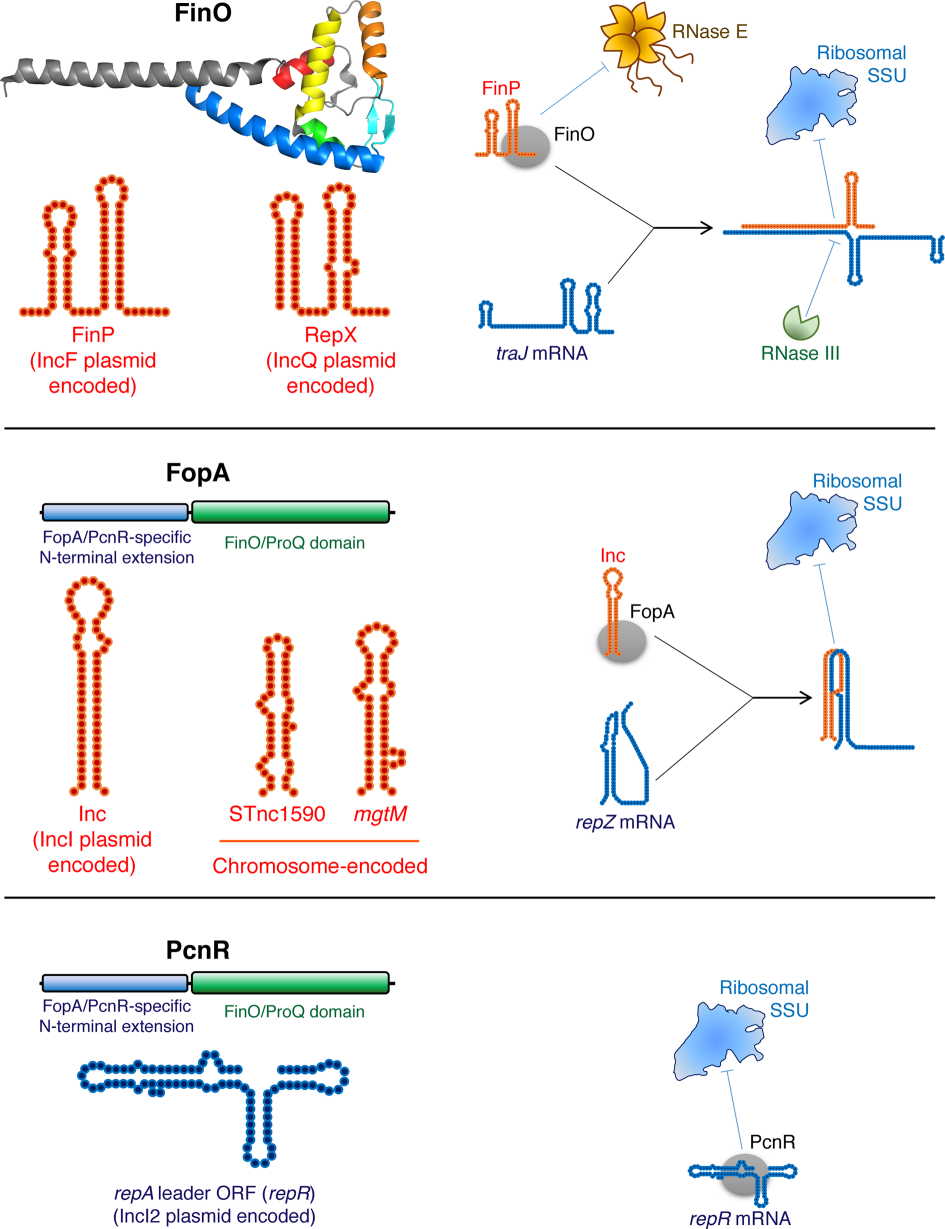
Characterised plasmid-encoded FinO/ProQ-family proteins For each protein, schematic structures of its main RNA ligands and a representative molecular mechanism of translational repression are shown (see the main text for detailed descriptions). Note that fine structural details of the shown interactions are currently unknown. The FinP-*traJ* and Inc-*repZ* duplexes are likely partial, even though the interacting RNAs are fully complementary to each other. FinO and FopA are primarily associated with the cognate sRNAs and may dissociate upon duplex formation. The conserved elements of the FinO/ProQ domain in the crystal structure of FinO (amino acids 33–184) [22] are coloured in the same way as for other proteins shown in Figure 2 (see also Figure 3B,C for their annotation). The structure is shown from the RNA-binding concave face. The FinO protein also has a disordered N-terminal extension, not shown here. The structures of FopA and PcnR have not yet been solved.

**Figure 2:**
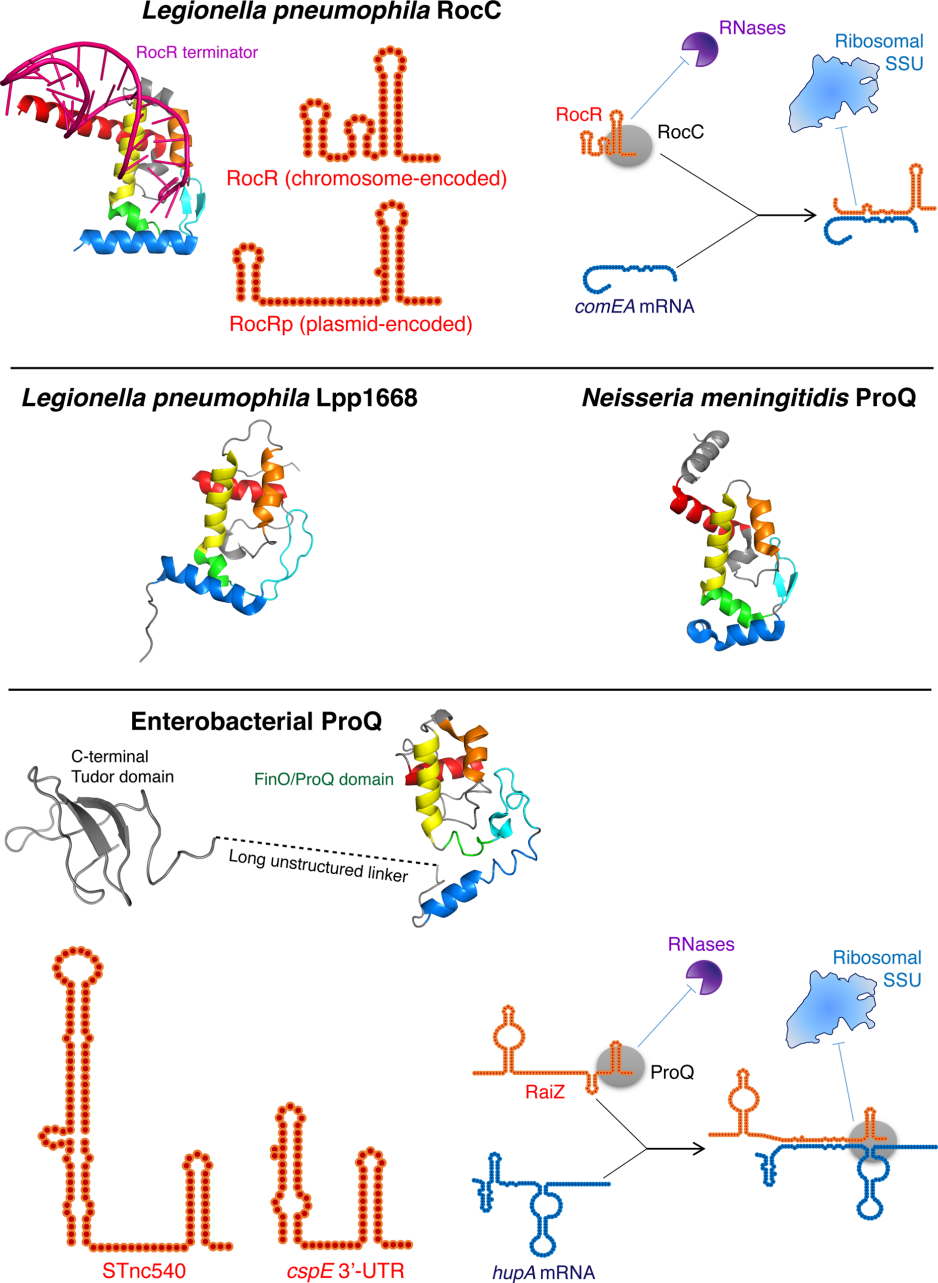
Characterised chromosome-encoded FinO/ProQ-family proteins For each protein, schematic structures of its select main RNA ligands and a representative molecular mechanism of translational repression are shown, wherever known (see the main text for detailed descriptions). As in the case of plasmid-encoded FinO-like proteins, structural details of the shown interactions are only partially understood. The conserved elements of the FinO/ProQ domain in the crystal structures of *L. pneumophila* RocC [63] (amino acids 11–125; in complex with the terminator domain of the RocR sRNA, magenta) and *N. meningitidis* ProQ (amino acids 1–124) [23] and in the NMR structures of Lpp1668 [25] and *E. coli* ProQ (amino acids 1–133 and 180–232) [24] are coloured in the same way for FinO shown in Figure 1 (see also Figure 3B,C for their annotation and a zoomed-in view of the RocC–RocR complex). The structures are shown from the concave face. RocC and *N. meningitidis* ProQ also possess unstructured C-terminal extensions, not shown here.

To date, half a dozen of FinO/ProQ homologues, covering a wide variety of origins and regulatory scopes, have been studied to an extent that permits us to connect their molecular mechanisms to biologically relevant macroscopic phenotypes. It seems appropriate first to briefly introduce the reader to the gallery of these proteins, which will be the main characters of the following sections (Figures 1 and 2).

### FinO

This prototypic member of the family is encoded on conjugative F-like plasmids of the IncF1 group widespread in *Escherichia coli* and related species [19]. FinO means ‘*Fertility Inhibition locus O*’, which reflects its main function: it represses the conjugation of its own plasmid. The mechanism involves a *cis*-encoded antisense RNA called FinP which base-pairs, with the help of FinO, to the ribosome-binding site of the *traJ* mRNA, encoding a key activator of the type IV pilus synthesis [22,26–29] (Figure 1). Without TraJ, the pilus operon cannot be induced, and conjugation becomes impossible. This of course limits the plasmid spread but also decreases the metabolic burden on the host cell and protects it from pilus-specific bacteriophages, which ensures the maintenance of F-like plasmids in the host cell population [30].

### FopA

FopA (‘*FinO domain protein on Plasmid/phage A*’) is encoded in the *Salmonella entreica* IncIα group colicin-producing plasmid pCol1B9, with closely related proteins in other enterobacteria (Figure 1). It accumulates in the stationary phase, specifically binds and stabilises the plasmid-encoded antisense RNA Inc and promotes its base-pairing with the *repZ* mRNA, encoding the replication initiator protein for pCol1B9 [31,32]. Inc binding disrupts a 5′-pseudoknot structure required for *repZ* translation initiation at a downstream ribosome-binding site. The repression of RepZ production by Inc/FopA permits pCol1B9 to auto-regulate its own copy number and decrease its fitness cost for the host [4].

### PcnR

Although phylogenetically close to FopA, the PcnR protein encoded on IncI2 group plasmids seems to exploit a totally different mechanism (Figure 1). It directly, independently from the *cis*-encoded antisense RNA, interacts with a stem-loop structure in the leader ORF *repR* just upstream of the *repA* gene, which specifies a key plasmid replication protein. As a result, RepA is not produced, and the replication of the plasmid is repressed, which helps to maintain its level at ∼1 copy/cell (hence the name ‘*Plasmid Copy Number Repressor’*). The PcnR-encoding plasmids often carry the colistin resistance gene *mcr-1*, associated with a high fitness cost in *E. coli* populations, which can be mitigated through the PcnR-mediated control of their copy number [20].

### RocC

RocC is one of the two chromosome-encoded ProQ homologues in *Legionella pneumophila* [25,33] (Figure 2). Its main role is repressing the natural competence of *Legionella* [33,34] (hence its name – ‘*Repressor Of Competence, RNA Chaperone*’). RocC stably binds and stabilises the RocR sRNA, which base-pairs in-*trans* with a few mRNAs encoding DNA uptake proteins (e.g. the dsDNA-binding protein ComEA and type IV pilus components), repressing their translation. This happens in the early exponential phase, where *Legionella* actively divides and incoming foreign DNA may endanger its genome integrity. By contrast, at the wake of the exponential phase, the acquisition of new DNA may confer adaptive advantages and improve survival: RocC and RocR expression shuts down, and the competence regulon gets activated [33]. The function of the second *L. pneumophila* ProQ-like protein, Lpp1663, is unknown [25].

### ProQ

The name ‘ProQ’ (‘*PROline uptake regulator Q*’) historically refers to one very specific phenotype of *E. coli* Δ*proQ* strains: the mutant bacteria were found to be deficient in the activity of the osmoregulatory transporter ProP, which is responsible for the uptake of proline and other compatible solutes [35]. However, recent comprehensive genome-wide studies in several γ- and β-proteobacteria revealed that the designation ‘ProQ’ actually corresponds to quite a disparate set of chromosome-encoded globally acting RNA chaperones with extensive suites of RNA ligands and a pervasive impact on bacterial gene expression [21].

*S. enterica* and *E. coli* ProQ were found to associate with ∼400 transcripts, including >50 sRNAs, and affect the expression of hundreds of genes involved in energy production, osmoregulation, amino acid metabolism, translation, biofilm formation, motility and virulence [3,36–39]. Enterobacterial ProQ directly stabilises many of its sRNA and mRNA ligands [3,36,40–44]. Similar to other members of the family, it is also extensively involved in sRNA-mediated regulation [37]. For instance, the ProQ-dependent sRNA RaiZ base-pairs with multiple targets [37], including the *hupA* mRNA that encodes the histone-like protein HU-α involved in genome compaction and global transcriptional control (Figure 2). RaiZ interacts with the ribosome-binding site of *hupA* and blocks its translation. ProQ critically contributes to this regulation by protecting RaiZ from cellular RNases and by stabilising the RaiZ-*hupA* duplex against the unwinding activity of the initiating ribosome [40]. Another example of ProQ-dependent sRNA is *S. enterica* STnc540 which represses, via direct base-pairing, the *mgtB* mRNA encoding a magnesium transporter important for the survival of the pathogen inside human cells [39].

In other Enterobacteriaceae (*Dickeya dadantii, Erwinia amylovora* and *Photorhabdus luminescens*), ProQ also seems to act as a general RBP and to be involved in many of the same physiological functions, such as osmolarity control, motility, biofilm formation, and virulence [45–47]. In the more distantly related *Pasteurella multocida*, ProQ was found to interact with ∼70 RNA species (mRNAs, sRNAs, tRNAs) and affect, mostly positively, the expression of ∼180 genes linked to carbohydrate metabolism and translation [48]. In the β-proteobacterium *Neisseria meningitidis*, a minimalistic ProQ homologue with RNA chaperone activity similarly interacts with ∼180 mRNAs and sRNAs and affects the transcript levels of >250 genes involved in multiple pathways, including energy production and amino acid metabolism [23,49].

## Evolution and spread of FinO/ProQ family proteins

**Figure 3:**
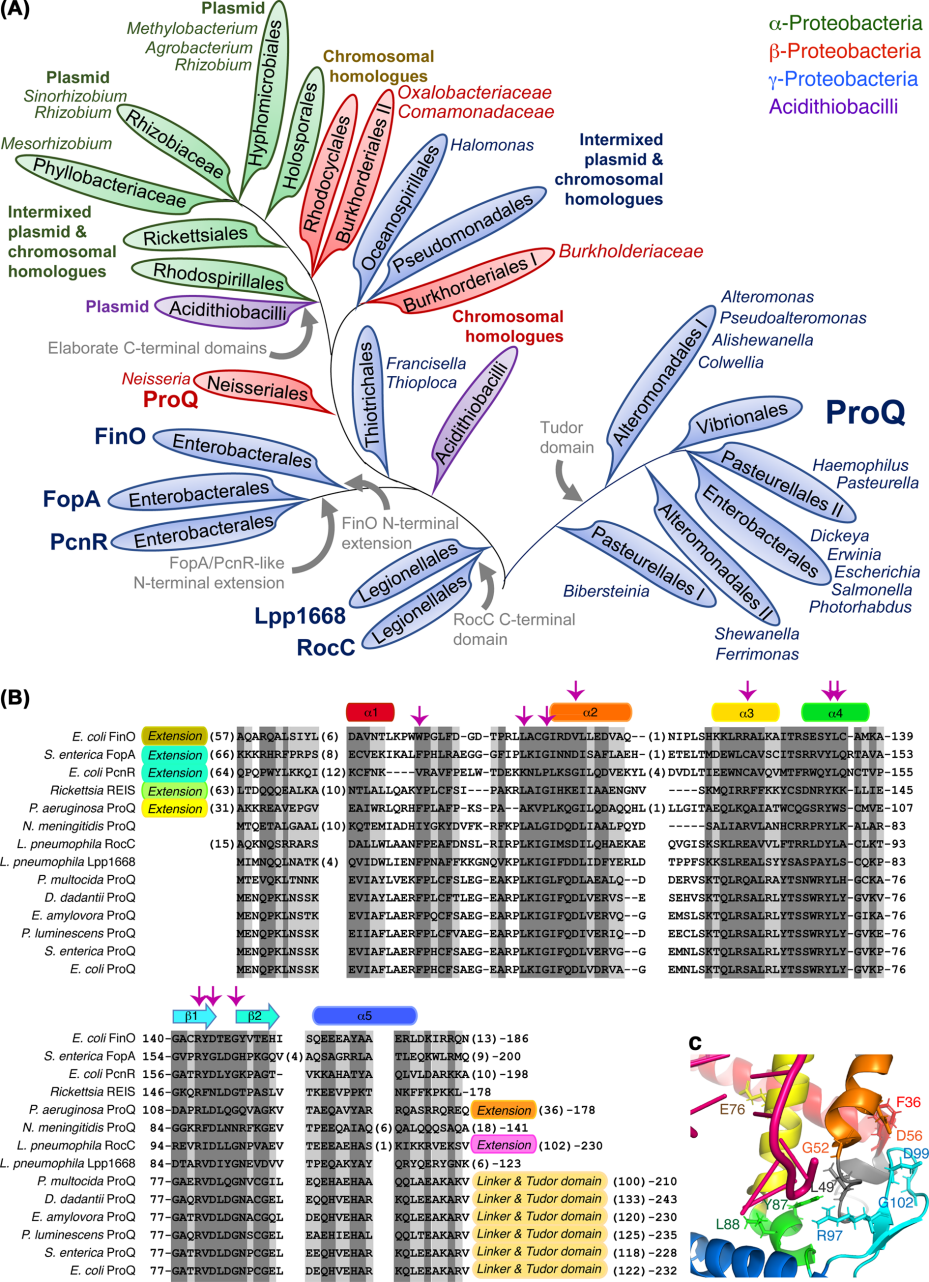
Genealogy of the FinO/ProQ family (**A**) Schematic tree showing the diversity and phylogenetic relationships between known FinO/ProQ homologues (adapted and modified from [3,33,50]). Grey arrows show major extra domain acquisition events. (**B**) Sequence alignment of core domains of the FinO/ProQ homologues reported in literature so-far (COBALT [110]). Dark grey shadowing corresponds to highly conserved residues, light grey, to moderately conserved ones. Structural elements are annotated above the alignment in the same colours as in Figures 1 and 2. The size of extensions is given in parentheses. Magenta arrows point at the residues which were repeatedly found to be required for RNA binding and/or stabilisation [24,25,33,42,43,59,61,64]. (**C**) Zoom-in view of the RNA-binding site of *L. pneumophila* RocC [63]. The 3′-end of the RocR sRNA is bound in a pocket formed by several conserved residues labelled in panel (B).

There are currently no systematic studies dedicated to the evolution of FinO/ProQ proteins. The little we know about the way they have spread throughout four Proteobacteria classes (α-, β-, γ-proteobacteria and Acidithiobacilli) comes from comparative analyses of a few thousand protein sequences and, importantly, of the physical location of their genes [3,33,50]. These studies broadly separated the family in two clades (Figure 3A). One is composed nearly exclusively of γ-proteobacterial chromosome-encoded ProQ proteins, which, as one can judge by several studies in Enterobacterales (*E. coli, S. enterica, E. amylovora* and *D. dadantii*) and Pasteurellales (*P. multocida*), are typically global RNA-binding regulators with a pervasive impact on cell physiology [3,37,45–48]. The other branch is extremely heterogeneous and loosely groups proteins encoded either on the choromosome, or on plasmids, or on bacteriophages, including the well-characterised enterobacterial plasmid repressors FinO [19], FopA [4], the *L. pneumophila* core genome-encoded competence repressor RocC [33], and the *N. meningitidis* global RNA chaperone ProQ [23,49].

The phylogenetic structure of the tree is not the same between the two branches. Whereas the former, ‘classical ProQ’ branch follows relatively faithfully the established phylogeny of the corresponding γ-proteobacteria, suggesting vertical inheritance, the latter, ‘eclectic branch’ is a mosaic of species with a more complex pattern. Somewhat apart, the two *L. pneumophila* chromosome-encoded FinO/ProQ proteins, RocC and Lpp1663, seem to arise from an early duplication event followed by paralogous evolution with both structural and functional divergence (see below) [25,33]. The plasmid-encoded FinO proteins form another well-delimited group within this branch. Encoded on F-like plasmids, they are horizontally spread genetic elements *par excellence*. The major role of FinO is believed to be repressing unnecessary production of the costly conjugative type IV pilus, which reduces the metabolic burden of the plasmid for the host and guarantees its maintenance in the bacterial population [19]. Other FinO-like proteins, such as *S. enterica* FopA and *E. coli* PcnR, repress the replication of the plasmids they are encoded in, which similarly contributes to the alleviation of their fitness cost [4,20]. It appears that FinO and FopA proteins also relatively easily cross inter-species barriers, since *E. coli* FinO-encoding plasmids have been found in some *Salmonella* isolates [51], and *Salmonella* can transfer its FopA-encoding plasmids to *E. coli* [52]. These properties enable both vertical and horizontal propagation of FinO-like genes among enterobacteria.

The rest of the ‘eclectic’ branch is a curious mixture of chromosomal and mobile DNA-encoded FinO/ProQ homologous with little respect for the phylogeny of their hosts and the location of their genes, suggesting a pervasive role for horizontal mechanisms in this clade. It appears that many a time genes specifying plasmid- or phage-encoded FinO/ProQ-like proteins were integrated into the bacterial chromosome. Notorious examples of this kind can be found in β-proteobacteria (*N. meningitidis*) [23] and especially in α-proteobacteria, where the implantation of the FinO/ProQ-family seems to be recent (Figure 3A). We detected 81 FinO/ProQ-like proteins in a total of 65 complete genomes coming from this class. Whereas in Rhizobiaceae essentially all identified homologues were plasmid-borne, Phyllobacteriaceae and Rickettsiaceae featured both chromosomal and plasmid representatives, sometimes co-occurring in the same cell. In most cases, they were very close to each other in terms of sequence homology. Moreover, the chromosomal FinO/ProQ genes were usually surrounded by mobility-related genes (specifying transposases, integrases, partition proteins, etc.) [18]. These observations strongly suggest that α-proteobacteria, much later than γ- and β-proteobacteria, started to ‘domesticate’ horizontally acquired FinO/ProQ-like proteins, which may, in the long run, result in their exaptation to serve core-genome post-transcriptional regulatory programmes. Some groups, e.g. the β-proteobacterial order Burkholderiales, have apparently evolved chromosome-encoded FinO/ProQ-like proteins several times independently, and some of these proteins are actually more closely related to homologues from the γ-proteobacterial order Pseudomonadales than to other β-proteobacteria.

Overall, FinO/ProQ-family proteins appear to combine vertical and horizontal spreading mechanisms. It is conceivable that their deep evolutionary roots are to be sought among selfish mobile genetic elements, where these proteins played narrowly defined functional roles directly related to their epidemic transmission and maintenance in bacterial populations. Once integrated into the chromosome, many of them likely suffered erosion as unnecessary in their new genomic context. This apparently happened with many plasmid-borne homologues in *S. enterica* and some *Rickettsia* species [53,54]. However, some FinO-like proteins might have been repurposed and integrated into host post-transcriptional networks as relatively specific regulators, like *Legionella* RocC [33], or as full-fledged RNA-binding hubs, like *Neisseria* and enterobacterial ProQ [21,49]. This process was likely helped by additional horizontal transfer events which, for instance, might have permitted γ-proteobacterial ProQ proteins to acquire a normally eukaryotic Tudor domain, critically important for their RNA chaperone activity [24,42,43,55]. Overall, steady broadening of the regulatory scope of FinO/ProQ homologues may be a major evolutionary vector within this protein family.

### FinO/ProQ family: the conserved

The wide diversity of functions and origins notwithstanding, all FinO/ProQ-like proteins share a few highly conserved, common traits that create the unique identity of this protein family [18,21]. All of them appear to be small (14–27 kDa), basic, and essentially monomeric proteins [22–25,56,57]. The latter property is relatively atypical among bacterial regulatory RBPs [1,15] (cf. dimeric CsrA, trimeric PNPase, tetrameric RNase E, hexameric Hfq and Rho) and may be linked to the unique mode of RNA recognition exploited by their diagnostic FinO/ProQ domain fold (Figures 1-3). This one is composed, with small variations, of five α-helices and a 2-stranded antiparallel β-sheet arranged in a very conserved way and forming a shallow ‘bowl’, which usually has an asymmetric charge distribution between its concave and convex surfaces, with large positively charged patches [18,22–25].

This unique fold turned out to be notoriously good at recognising and strongly binding (often with low-nanomolar *K*_d_s) structured RNA molecules where a helical element of at least two base pairs is obligatorily followed by a single-stranded 3′-tail [3,23,25,36,38,40,55,58–61]. Such structural elements are naturally rife in intrinsic terminators of bacterial transcripts. Indeed, most known RNA partners of FinO/ProQ-family proteins engage the protein via their terminator structures (Figures 1 and 2), which was confirmed by both focused studies *in vitro* and by global interactomic profiling of ligands *in vivo* [4,33,36,37,39,40,46,48,49,58]. However, a few prominent processed ProQ ligands, such as certain phage-encoded and intragenic sRNAs, are devoid of terminators and appear to interact via other similarly structured elements [3,36,62].

The striking dependence of the FinO/ProQ domain interactions on this specific structural pattern within their RNA ligands was observed already in early studies of FinO and ProQ and more recently in works on *L. pneumophila* homologues [3,23,25,36,40,58–61,63]. Cross-linking, mutagenesis, hydrogen deuterium exchange, and NMR studies identified critical residues involved in RNA binding and regulation, many of which turned out to be highly conserved (Figures 1-3). These studies strongly implicated the positively charged concave surface of the FinO/ProQ domain, and in particular the helix α4 and the β-sheet, as the main platform for a high-affinity interaction [24,25,33,42,43,55,61,64]. Decisive insights into how such an unusual recognition mechanism is brought about have been recently obtained thanks to the first crystal structure of a FinO/ProQ-family protein, RocC, in complex with its native RNA partner RocR [63]. The RocC protein interacts with the second, terminator stem-loop of RocR but, surprisingly, does not form extensive contacts with the double-stranded portion of the ligand (Figure 2). Instead, its positively charged binding pocket on the concave side of the FinO/ProQ domain cradles the single-stranded 3′-tail of the terminator, which is wound in an A-form helical conformation as if it followed through the shape of the stem it emerged from (Figures 2 and 3C). Shortly before the 3′-end, the tail makes a U-turn, which is specifically recognised by conserved residues in the binding pocket. Importantly, the last nucleoside must have a free 3′-hydroxyl group in order to fit into the pocket, the presence of phosphates on the 3′-end not being tolerated, as was also observed for FinO [59,63]. The RNA recognition appears to be largely sequence-independent: the FinO/ProQ proteins only seem to read the shape of the ligand, using strategically positioned lysines and arginines interacting with the sugar-phosphate backbone [24,36,42,43,49,58,59,61,63,64,110]. However, *L. pneumophila* Lpp1663 does seem to show clear preference for 3′-poly(U) tails [25]. The combination of these factors may explain why FinO/ProQ-family proteins often prefer intrinsic terminators as binding sites. It also echoes the strategy of RNA recognition by a fellow RNA chaperone, Hfq, which similarly senses the 3′-hydroxyl of the intrinsic terminator to form a stable complex with sRNAs via its proximal face [65,66] (we will discuss the ‘conflict-of-interest’ arising due to this similarity between the two RNA chaperones in the section ‘*Relations with other global RNA-binding proteins*’).

Despite the immense diversity of ligands, most known FinO/ProQ proteins stably associate with at least one highly structured base-pairing sRNA which, by interacting at or close to the ribosome-binding site, negatively regulates the translation and/or stability of its target mRNAs (Figures 1 and 2). The role of FinO/ProQ proteins in such regulatory events is usually two-fold: (i) they stabilise their sRNA ligands against cellular RNases and (ii) they act as RNA chaperones by loosening folded RNA domains and promoting the sRNA-mRNA base pairing.

The stabilising role of FinO/ProQ proteins, conferred directly by the FinO/ProQ domain [42,43], is very important for the functional output of the partner RNA. Thus, the FinO-mediated shielding of the FinP sRNA from an RNase E attack allows for efficient repression of its *traJ* mRNA target [67,68]. Outside a base-pairing context, enterobacterial ProQ sitting on the terminator of the *cspE* mRNA shields it from the 3′-exoribonuclease RNase II [36] and *N. meningitidis* ProQ can protect the *rmpG* mRNA from PNPase-mediated degradation *in vitro* [49]. A general protective role of ProQ has been observed genome-wide in *S. enterica* [36], *P. multocida* [48] and* N. meningitidis* [49].

The RNA chaperone properties of FinO/ProQ-like proteins seem to be omnipresent in this family and have been quite well-characterised [4,23,55,69]. Unlike Hfq-dependent sRNAs, which base-pair with their targets via single-stranded regions [2], many characterised cases of sRNA–mRNA interactions mediated by FinO/ProQ proteins involve stem-loop structures from both partner RNAs [4,26,40]. The seeding interaction is thought to occur between short single-stranded nucleotide stretches (e.g. kissing loops) and gradually propagate to the more structured neighbourhood [28,29,31,32,40,70]. This interaction mode, quite usual among *cis*-encoded antisense RNAs [71], could be greatly facilitated by an RNA chaperone. Indeed, FopA increases by 7-fold the base-pairing rate between the Inc sRNA and the *repZ* 5′-UTR to repress the replication of the pCol1B9 plasmid it is encoded on [4]. In a similar mechanism, FinO considerably accelerates the FinO-*traJ* mRNA interaction, which is normally required for the conjugation-repressing function of FinP *in vivo* [22,26–29,69]. Interestingly, the ProQ protein from the β-proteobacterium *N. meningitidis*, which normally cannot encounter enterobacterial F-plasmids in nature, was found to be able to accelerate the FinP-*traJ* mRNA base-pairing and repress the conjugative transfer of the F-plasmid, when ectopically supplied in *E. coli* [23]. This highlights the partially interchangeable nature of the RNA chaperone activity among FinO/ProQ proteins. However, as will be discussed in the following sections, the origin of this activity is likely paraphyletic, associated with non-conserved domains, and may instead reflect convergent evolution between various FinO/ProQ-family members.

### FinO/ProQ family: the divergent

‘A regulatory chaperone transacting structured RNA with the help of a conserved, mostly helical domain’ – this is probably just as far as one can go with one's assumptions regarding a newly encountered FinO/ProQ-family protein. Beyond this generic image begin endless structural, biochemical and functional variations, reflecting rapid radiation from a primordial core, which likely resembled a plain, single-domain and relatively low-affinity RBP similar to *Legionella* Lpp1668 (Figure 2) [25].

The first striking feature one notices looking at the FinO/ProQ family is the diversity of their interactomic and regulatory scopes. All known plasmid-encoded representatives are strictly specialised and have, with rare exceptions, only one mRNA and/or sRNA target [4,20,72]. Although all of them typically act as negative regulators of their own plasmids, the molecular mechanisms and the biological processes they target seem to be divergent. FinO, programmed with the sRNA FinP, represses conjugation of IncF group plasmids, such as the *S. enterica* pSLT [19,72]. However, the same *S. enterica* FinO protein, when reprogrammed with the foster sRNA RepX (Figure 1), cross-regulates the replication of a cohabitating IncQ group plasmid pRSF1010, indicating that, in function of context, it can target different genetic processes [72] (this interesting case will be discussed in more detail in the section ‘Multiple FinO/ProQ proteins in one cell: *ménage à trois*’). FopA and PcnR also curtail the replication of their respective plasmids, but through different mechanisms [4,20] (Figure 1).

Chromosome-encoded homologues are even more dissimilar in this respect. The regulatory scope of *Legionella* RocC is overall analogous to that of plasmid-encoded FinO-like proteins. It associates nearly exclusively with one sRNA, RocR, which base-pairs with four mRNAs implicated in competence control. However, the RocR sRNA is encoded in-*trans* and not in-*cis* with respect to its targets, as in the case of plasmid FinO-like proteins [33]. Interestingly, just like in the above-mentioned *Salmonella* FinO case, RocC can associate with a foster, pLPL plasmid encoded sRNA called RocRp (Figure 2). RocRp guides RocC to the same mRNA targets as RocR, but this happens outside the usual time frame of RocR action, which provokes constitutive repression of competence. This apparently selfish mechanism may permit the plasmid to ward off competing mobile genetic elements [73]. It shows that the border between plasmid and chromosomal RNA regulation may be very thin as both can rely on shared FinO/ProQ-like proteins.

ProQ proteins from γ-proteobacteria and *Neisseria* are at the opposite extreme in this spectrum: they are global regulators with hundreds of targets, implicated in multiple physiological pathways. However, the exact targets and regulons of these proteins may be quite different from one species to another. For example, *E. coli* and *D. dadantii* ProQ proteins are involved in resistance to osmotic stress [45,74], but this role is not conserved in *P. multocida* [48]. Whereas in *E. coli* and *P. luminescens* ProQ positively regulates biofilm formation [38,47], its effect is opposite in the closely related *E. amylovora* and *D. dadantii* [45,46], and null in the phylogenetically distant *N. meningitidis* [49].

The sources of this functional divergence are likely both *extrinsic* (i.e. linked to differences in transcriptomes between species and even between growth conditions for the same species [3,36,37,39,46,48,49,72]) and *intrinsic* (i.e. stemming from the proteins themselves). Although FinO/ProQ-like proteins share the same diagnostic domain as the main RNA-binding platform, its structure is variable from one protein to another, which likely has a direct impact on what each of them can or cannot do (Figures 1 and 2). In general, it appears that the degree of ‘openness’ of the FinO/ProQ domain and the exact pattern of positively charged residues across its two faces define, to a large extent, the interactomic scope of these proteins (see for illustration Figure 1B) in a nice review by Olejniczak and Storz [18]). In FinO, the positive patch is largely confined to the concave side, and α5 together with the long N-terminal helix strongly constrain the potential RNA-binding surface, which may explain why FinO is a highly specific RBP, binding nearly exclusively to FinP [22,56,72] (Figure 1). By contrast, in *E. coli* and *N. meningitidis* ProQ, the same domain is very accessible: its ‘framing’ helices α1 and α5 are swung open (Figure 2), and the positively charged residues extend on both sides of the bowl forming a kind of ‘saddle’, permitting the accommodation of RNA ligands with varied shapes [23,24]. If this intuition is correct, the functionally uncharacterised *Legionella* ProQ homologue Lpp1668, which has a similar anatomy, may have a relatively broad suite of RNA ligands [25]. It will be exciting to test this prediction in a direct interactomic study.

An important feature recognised by the FinO/ProQ domains and likely contributing to their binding specificity is the length and the composition of the RNA 3′-terminal tail. The optimum here seems to differ between homologues. FinP must have at least 5–6 single-stranded nucleotides on its 3′-terminus to be strongly bound by FinO [58,72]. By contrast, FopA must do with as few as 3–4 loosely paired nucleotides on the 3′-ends of its ligands [4,31] (Figure 1). The FinO/ProQ domain of *E. coli* ProQ normally requires between 4 and 9 nucleotides; both shortening or extending the tail beyond these limits results in decreased affinity [60].

Another critical factor contributing to the structural and functional diversity of FinO/ProQ-like proteins is extensions and additional domains. Given their extremely dissimilar nature, they appear to have been acquired independently in each lineage (Figure 3A). Additional structural elements may restrict the access to the FinO/ProQ domain, as likely happens in FinO [22], or, on the contrary, offer additional landing pads to enable interactions with a larger number of transcripts, which do not necessarily fit the ‘structural code’ of the FinO/ProQ domain. This ‘extra-binding’ role is best characterised on the example of enterobacterial ProQ, where a unique C-terminal Tudor domain, connected to the N-terminal FinO/ProQ core by a long unstructured linker, significantly contributes to interactions with some but not all ligands [24,38,42,60,64]. It is conceivable that the very long (on the order of ∼10 nm) linker enables ProQ to sample and bridge RNA regions that are otherwise too far from the 3′-end anchored in the FinO/ProQ domain (Figure 2).

It seems that the major molecular functionality that extensions bring in the FinO/ProQ-like proteins is RNA chaperone activity. Inherently flexible, charged or possessing exposed hydrophobic residues, these structural addenda often catalyse RNA duplexing and strand exchange, while the FinO/ProQ domain *per se* is usually inert in such reactions, only providing high-affinity binding and stability [25,33,55,59,69]. Furthermore, a number of studies demonstrated an inverse relationship between the binding strength and the RNA chaperone activity in many FinO/ProQ-family proteins [33,42,55,69]. This interesting observation suggests that the FinO/ProQ domain and the extensions are responsible for equally valuable but biochemically conflicting functions within the same RBP.

**Figure 4:**
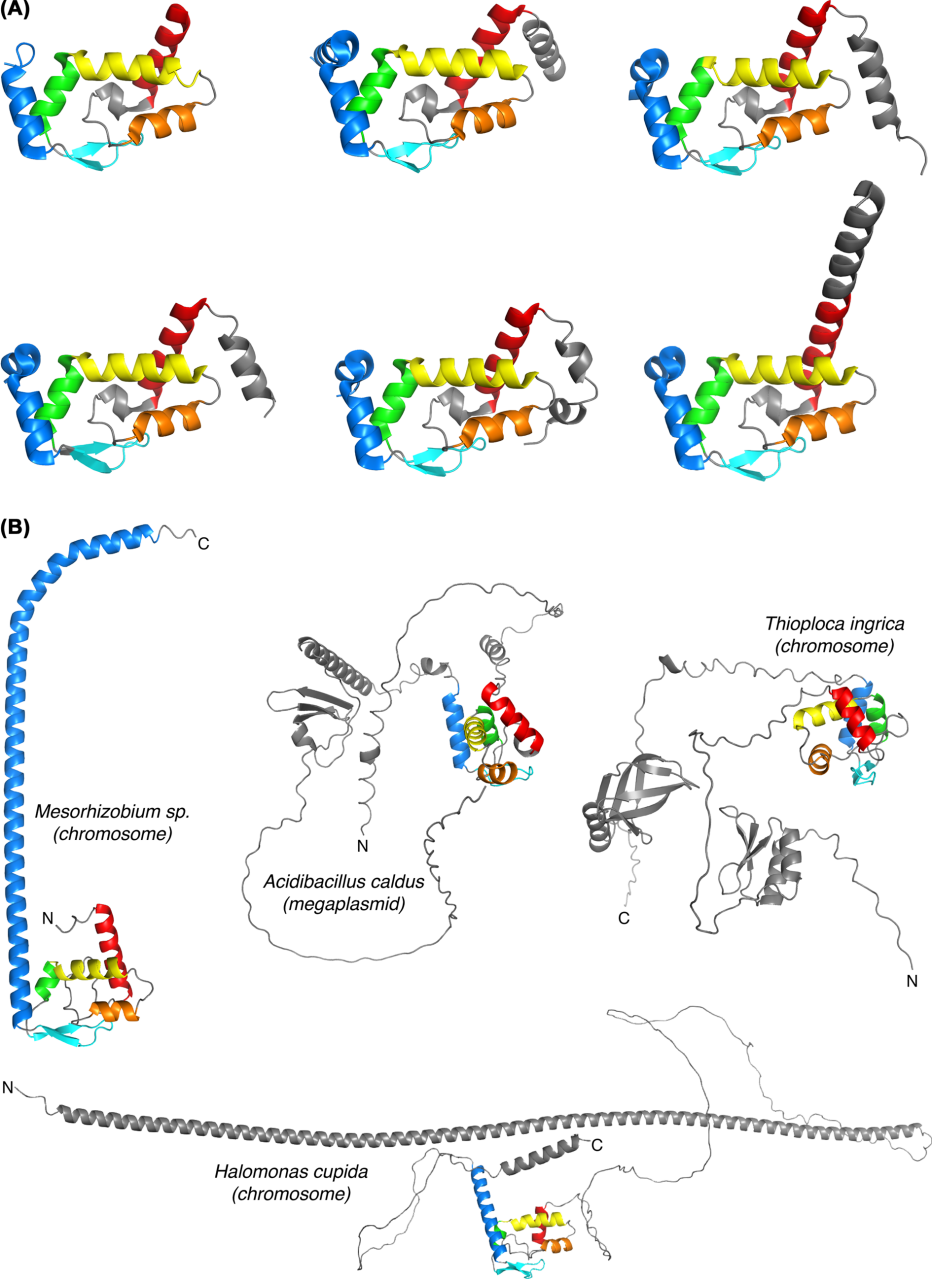
Mobile extensions of FinO/ProQ-like proteins (**A**) The crystal structure of *N. meningitidis* ProQ contains 6 chains in the crystallographic asymmetric unit [23]. Comparison of these individual chains permits to assess the conformational heterogeneity in this protein. The folded core of the protein (formed by the helices α2, α3 and α4, the β-sheet, and the connecting loops) is invariant and likely rigid. By contrast, the helix α1 (red and grey) shows high flexibility, permitting it to sample a variety of conformations. In the first chain (top left), the flexibility of both helices α1 and α5 (blue) is so high that it does not permit to see their extremities at all. (**B**) Examples of highly complex FinO/ProQ-like proteins with additional folded and/or disordered domains (as predicted by AlphaFold [111]), mined from genomes of understudied proteobacterial groups.

The mobility of extensions is apparently very important for the RNA chaperone activity. We already mentioned the very long unstructured linker in enterobacterial ProQ [24]. Another nice visual example of how dramatically an extension may change its conformation can be observed in the crystal structure of *N. meningitidis* ProQ, where the N-terminus, including α1, is folded differently in every chain of the asymmetric unit [23] (Figure 4A). Even the long N-terminal helix of FinO (Figure 1) is not rigid and can bend toward the FinO/ProQ core of the protein as it interacts with FinP and the *traJ* mRNA [61].

The additional domains are extremely important for the *in vivo* functions of FinO/ProQ proteins. Mutagenesis studies indicated that without the linker and the Tudor domain, primarily responsible for the RNA duplexing and strand-exchange activities, enterobacterial ProQ can bind RNA but fails to drive regulatory events [42,43]. Similarly, the FinO/ProQ domain of RocC stabilises the RocR sRNA *in vivo* but does not repress competence, indicating that the long C-terminal extension of this protein is functionally essential [33]. Mining the UniProt database [75,111] for other, so-far uncharacterised, members of the FinO/ProQ family revealed the existence of sometimes exuberantly complex proteins with yet other kinds of additional domains and likely new exciting biological properties (Figure 4B).

### FinO/ProQ family: the convergent

The wide diversity of additional domains and unstructured regions driving RNA chaperone-related activities in different FinO/ProQ-like proteins represents a striking case of convergent evolution toward the same biochemical function. Convergence is also manifested at the level of some physiological functions of the FinO/ProQ family. We already discussed that plasmid regulation by FinO, FopA and PcnR, although mechanistically different, has a largely similar outcome permitting them to keep a low profile in the bacterial population [4,19,20,72]. Chromosome-encoded ProQ proteins from several pathogenic bacteria are strongly involved in virulence, and given the diversity of ecological niches and underlying genomic elements, such a role must have evolved independently multiple times [39,45,49]. Furthermore, ProQ seems to be recurrently associated with translation, albeit through different mechanisms. It interacts with ribosomes in *E. coli, S. enterica* and *Pseudomonas aeruginosa* [3,38,76,77]. In *P. multocida* and *N. meningitidis*, ProQ binds and often regulates tRNAs and mRNAs encoding ribosomal proteins [48,49]. In *S. enterica*, during the exponential phase, the ProQ interactome is largely dominated by rRNA leader sequences, suggesting a link to ribosome biogenesis [3,72].

However, the most pervasive functional association, shared by most studied proteins of the FinO/ProQ family, is DNA metabolism and genome integrity (see Figure 6, below). In addition to the repression of mobile and selfish genetic elements (plasmids, phages, transposons, toxin–antitoxin loci, and genomic islands), various FinO/ProQ-like proteins participate in competence control [50], bind and regulate multiple mRNAs related to genome compaction, recombination, protection against oxidative and genotoxic stress [3,49]. The already mentioned enterobacterial sRNA RaiZ regulates the major DNA-compacting histone-like protein HU-α [40]. Interestingly, mRNAs of HU-α and of the other general DNA-binding protein IHF are prominent ProQ targets not only in enterobacteria but also in the phylogenetically more distant *P. multocida* [3,37,40,48]. Another conserved enterobacterial ProQ-dependent sRNA, SraL, bridges its translation- and DNA metabolism-related regulons in *E. coli*. It represses the expression of ribosome-associated trigger factor and up-regulates Rho, which protects the genome from spurious transcription leading to R-loops and dsDNA breaks [41,78,79]. With all this in mind, it is not surprising that Δ*proQ* strains are often sensitive to DNA-damaging agents, oxidative stress, and translation-targeting antibiotics [3,49,80,81].

The above-described diversity of dissimilar regulatory mechanisms has apparently arisen independently in different bacteria. It makes the recurrent implication of FinO/ProQ-like proteins in key genetic processes at the level of DNA and translation especially intriguing and warranting more detailed mechanistic studies.

### Multiple FinO/ProQ proteins in one cell: ménage à trois

**Figure 5:**
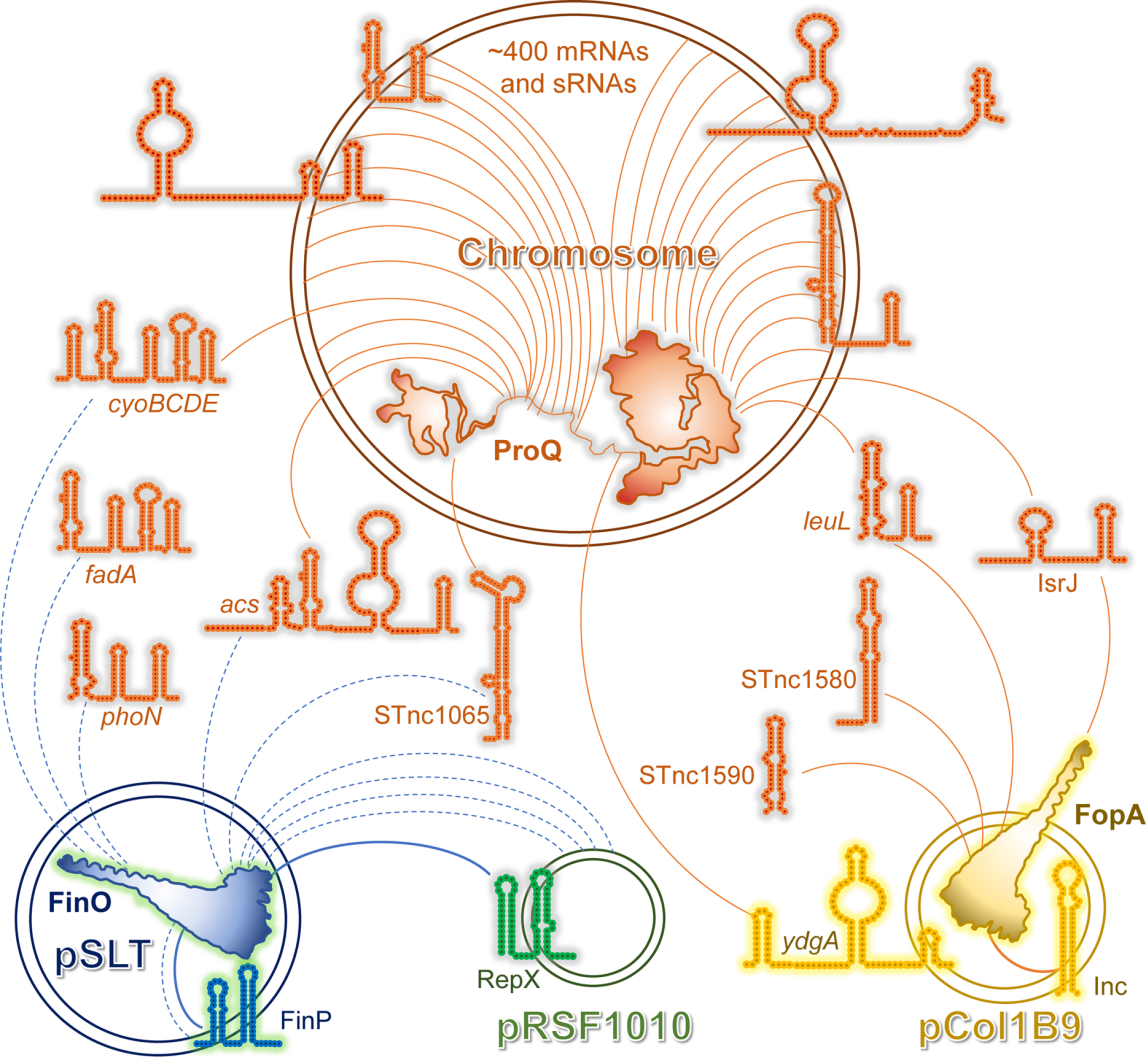
Replicons and FinO/ProQ-family proteins of *Salmonella* Typhimurium SL1344 Colour code for the RNA species and proteins corresponds to their replicon of origin. Examples of RNA ligands bound by one or several FinO/ProQ-like proteins are provided based on genome-wide interactomic studies [3,4,72]. Prevalent interactions are shown with full lines, whereas lowly populated, occasional binding is rendered in dotted lines.

A situation both rare and curious from the point of view of regulation and evolutionary implications occurred in some *Salmonella* Typhumurium strains, such as SL1344 [82]. This pathogenic enterobacterium possesses four replicons and three FinO/ProQ-family proteins in one cell: chromosome-encoded ProQ [3], pSLT plasmid encoded FinO [72] and pCol1B9 plasmid encoded FopA [4], while the third plasmid pRSF1010 does not encode any FinO/ProQ-like homologue (Figure 5). In previous sections, we discussed the role of each protein in autoregulating its plasmid of origin. Here, we will dwell a bit on the intricate relationship between them and the respective regulons.

**Figure 6:**
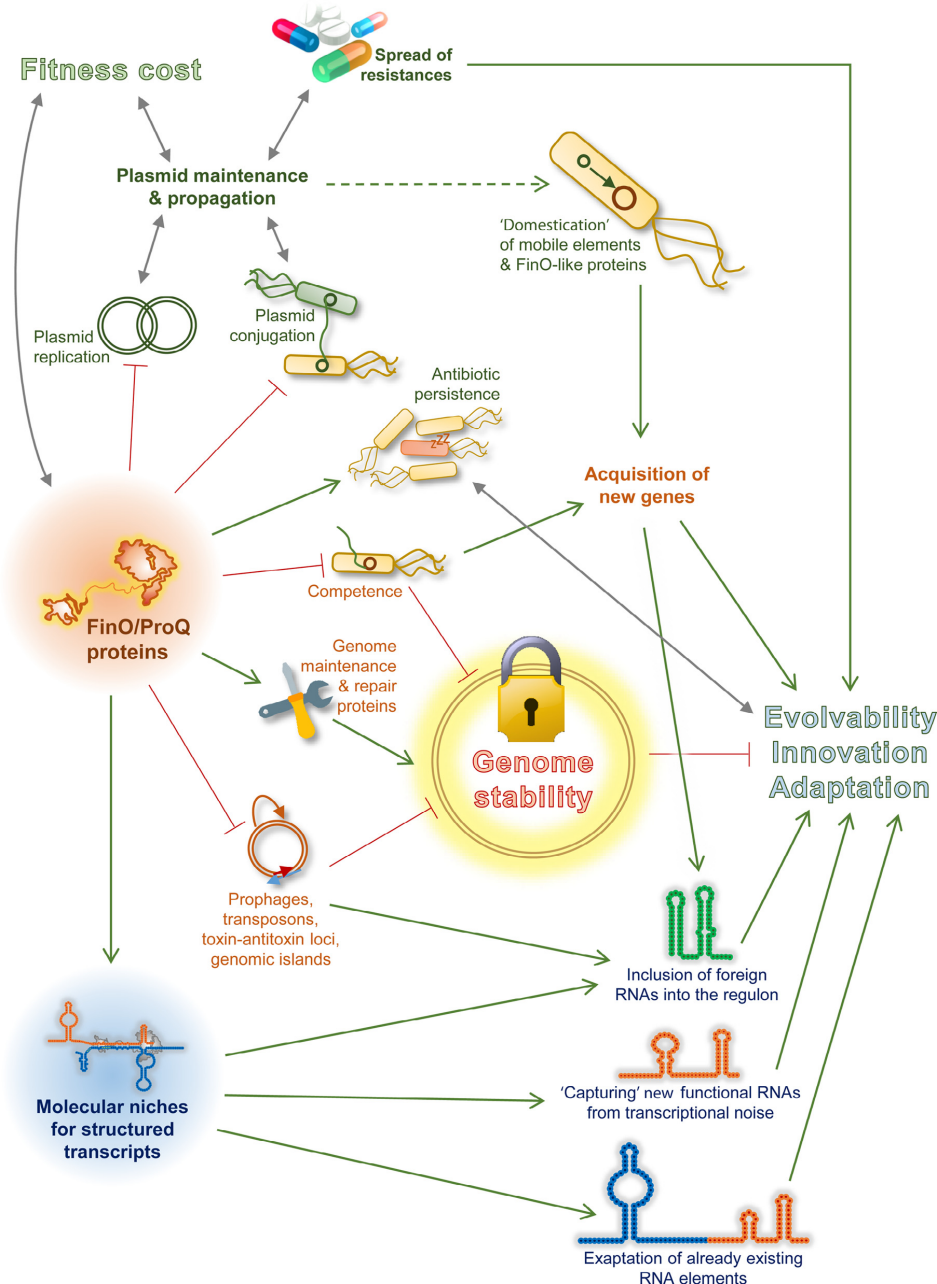
Evolutionary implications of FinO/ProQ-family proteins Both negative and positive molecular mechanisms mediated by FinO/ProQ-like proteins contribute in various ways to the evolution of their bacterial hosts. By repressing the replication or conjugation of their own plasmids, FinO-, FopA- and PcnR-like proteins seemingly limit their propagation in bacterial populations, but at the same time they maximise the chances of their maintenance by mitigating the fitness cost to the host. This may have far-reaching ecological and medical consequences, since such mechanisms ensure the spread of associated antibiotic resistances and virulence genes. Chromosome-encoded ProQ homologues can also be associated with significant fitness costs as they support energetically expensive processes (e.g. flagella, T3SS synthesis, translation). However, under some conditions (exposure to antibiotics, infection), they ameliorate the survival chances of bacteria, by contributing to antibiotic persistence and pathogenicity. FinO/ProQ family proteins also play important roles in genomic innovation. Eventually, plasmids and other mobile elements get ‘domesticated’ and their genes, including FinO-like proteins, integrated into the regulatory circuitry of the host. The acquisition of new genes is also regulated by RocC-like systems through the competence control. While this process can drive genome evolution, the failure to limit foreign DNA uptake may impact on the genome stability. Endogenously, ProQ proteins in several species positively regulate proteins involved in genome maintenance and repair and limit the expression of selfish DNA elements, thereby also contributing to the genome stability. In a long-term perspective, FinO/ProQ-like proteins create molecular niches that enable the recruitment of new structured RNAs into post-transcriptional regulatory pathways of the host bacterium. Such new RNAs can be obtained through horizontal transfer, captured from transcriptional noise, or exapted from pre-existing core genome- or genetic element-encoded functional transcripts.

There is no evidence that the three proteins are anyhow connected in terms of origin, and it is almost certain that such an unusual configuration was brought about by a horizontal transfer of plasmids from other bacteria into the *Salmonella* host, who already had a chromosome-encoded ProQ. Such a transfer has certainly broadened the genomic repertoire of *Salmonella* and had significant ecological consequences: pSLT carries the virulence-associated *spv* operon [83], pCol1B9 encodes colicin Ib [52], whereas pRSF1010 confers resistance to streptomycin and sulfonamides [84]. But it also introduced a significant metabolic burden for the host cell and created a regulatory conundrum where three RNA chaperones with conceptually similar binding modes, bathing in a cytosol rife with potentially suitable RNA ligands, must carefully delimit their interactomes and regulons to avoid deleterious crosstalk. Even though plasmid-encoded FinO-like proteins are reputed to be highly specific, likely thanks to their N-terminal extensions that may guard access to the RNA-binding site (see previous sections), the challenge remains tall for ProQ, which is very abundant and inherently promiscuous and could more easily step on a strange regulatory territory.

Strikingly, interactomic studies performed on each FinO/ProQ homologue in this system showed that they discriminate their cognate RNA ligands with exquisite selectivity and only in rare cases accept transcripts arising from cohabitating replicons. Thus, ∼80% of RNA bound by FopA is its cognate Inc sRNA [4]. Between 80% and 95% of the FinO molecules are occupied by the cognate FinP sRNA. However, the remaining 5–20% of the FinO pool binds to a foster sRNA, RepX, a structural mimic of FinP produced from pRSF1010 (Figure 1), and to a few mRNAs from the same plasmid. FinO stabilises RepX and may help it to repress the *repA* mRNA encoding the pRSF1010 replication helicase. Thus, FinO fully included pRSF1010 into its ‘zone of influence’ [72]. Biological consequences of this entanglement are not entirely clear. pRSF1010 is a typical IncQ group parasitic mobilisable plasmid that exploits cohabitating conjugative plasmids to spread across bacterial population. But IncF group plasmids, such as pSLT, actively exclude pRSF1010 during conjugation, suggesting that pSLT rather tends to interfere with pRSF1010 than to help it [85]. Therefore, FinO repressing the pRSF1010 replication might be just another such interference mechanism. RepX can apparently repress *repA* in the absence of FinO [86], suggesting that the cohabitation of pRSF1010 and pSLT is not strictly required to maintain the former in the bacterial population. However, co-opting FinO to more tightly down-regulate pRSF1010 replication may be advantageous for both plasmids, given that their cumulative fitness cost is high and potent repressive mechanisms are required to keep their copy number to as low a level as possible. This might explain why RepX does look to be selected for binding to FinO [72].

Both FopA and FinO were also found to occasionally bind chromosome-encoded sRNAs and mRNAs (Figure 5). While such transcripts constitute but a tiny fraction of the FinO interactome [72], FopA does seem to engage significantly with a few genome-derived RNA species that mimic its main ligand Inc [4] (Figure 1). It is conceivable that FopA may steadily expand its regulatory territory by including foreign RNA elements and increasingly cross-wire with the core genome, reinforcing the integration of pCol1B9 into the complex genetic system of *Salmonella*. Intriguingly, while many chromosome-encoded transcripts bound by FopA and FinO are also bound by ProQ (probably due to the similarity of their recognition codes), ProQ practically does not interact with plasmid-encoded RNAs (with the exception of the pCol1B9-encoded transposase *ydgA*) [3,72]. Therefore, ProQ remains devoted to chromosomal transcripts, reflecting the very advanced degree of ‘domestication’ of this FinO/ProQ-family representative. Similarly, FinO does not interact with pCol1B9-derived RNAs [72], and *vice versa* FopA does not bind pSLT or pRSF1010 transcripts [4] – they do not step in each other's realm.

Overall, the three FinO/ProQ homologues manage, by so-far obscure means, to delimit their regulatory territories, but some interesting tectonic shifts and intriguing overlaps have already begun to appear, making this *Salmonella* system an exciting real-time evolutionary laboratory.

## Relations with other global RNA-binding proteins

FinO/ProQ-like proteins are not alone to deal with RNA in the cell and must cope with the presence of many other RBPs [1,15]. Proteins are subject to natural selection just as organisms are, and the evolutionary survival of FinO/ProQ proteins largely depends on the definition of their molecular/regulatory niche and their ability to compete or collaborate with other RBPs existing in the system and doing similar things. The competition is tough. Choosing to bind to intrinsic terminators means entering a territory already disputed by 3′-exoribonucleases and the other major RNA chaperone Hfq [65,66,87]. In this section we will briefly discuss how FinO/ProQ-family proteins establish their relationships with some major, conserved, global RNA-binding regulators.

RNA binding by FinO/ProQ proteins typically results in its stabilisation *in vivo*, implying an antagonistic relationship with degradative RNases. Indeed, FinO protects FinP from RNase E-mediated turnover [68], and as much as a third of all ProQ-binding sites in *S. enterica* overlap with RNase E-targeted regions within the same RNA species, suggesting general RNA protection by FinO/ProQ proteins against this major bacterial degradative endoribonuclease [36,88]. At the same time, RNase E generates a number of processed ProQ-dependent sRNAs, such as RaiZ, from mRNA precursors and probably contributes to the turnover of their targets upon base pairing and translation repression, thereby concurring with ProQ-mediated riboregulation [40,62,88].

The relationship with the dsRNA-specific endoribonuclease RNase III is also nuanced. This enzyme degrades FinP-*traJ* duplexes, thereby completing FinO-mediated regulation [68], and it likely plays a similar role in a number of other mechanisms involving ProQ-dependent *cis*-encoded antisense RNAs forming perfect duplexes with their targets [3,89,90]. On the other hand, due to the affinity of ProQ toward dsRNA, one can expect situations where ProQ would prevent RNase III from accessing the duplex. One such case has been described in *E. coli*, where ProQ binding rescues the duplex between the Hfq-dependent sRNA RybB and its sponge RbsZ from RNase III-mediated turnover [37].

As already mentioned, by binding at terminators ProQ shields some of its ligands from 3′-exoRNases, such as RNase II [36] and PNPase [49], although this mechanism may not be general [37]. Nevertheless, a recent elegant hypothesis proposed that one of the reasons why RNA chaperones like ProQ and Hfq failed to find their way in Gram-positive and some Gram-negative bacteria is the presence of 5′-exoribonuclease activity supplied by RNase J, which makes these 3′-end protecting RBPs largely useless in such a context [91].

This brings us to the most intensely discussed interplay between ProQ and Hfq, which is especially well studied in enterobacteria [15,18]. These two global RBPs have much in common. Both interact with Rho-independent terminators and similarly require a 3′-hydroxyl for high-affinity binding [59,65,66]. Both have a significant stabilising effect on their ligands. Both are RNA chaperones, interact with sRNAs and mRNAs and promote their base-pairing to regulate mRNA translation and/or stability [92,93]. A conflict seems unavoidable. So how do they delimit their regulatory territories?

Part of the response comes from their intrinsic binding specificities. While ProQ prefers structured RNAs, Hfq typically associates with largely single-stranded transcripts and, unlike ProQ, does not require stem-loops for stable binding [60,94]. Another important discriminating feature: while Hfq needs a single-stranded 3′-terminal poly(U) tail of at least 6 nt (one uridine per Hfq monomer [65,66]), ProQ can do with as few as 4 nt [60]. Indeed, the 3′-terminal tails of ProQ-bound transcripts are statistically shorter than those of Hfq-associated RNAs [3,36]. To enforce discrimination between Hfq and ProQ, a region just upstream of the terminator stem-loop may be implicated. It is often U-rich in Hfq-dependent sRNAs, which prevents the 3′-poly(U) tail from base pairing and additionally provides an extra binding site for the rim of the Hfq hexamer [95,96]. By contrast, in many ProQ-dependent sRNAs this region is A-rich, which may transiently sequester the 3′-poly(U) tail in a weak base-pairing interaction, thereby excluding Hfq from binding [60].

These discriminatory measures, along with other still poorly understood factors, do seem to work, since the *in vivo* Hfq and ProQ interactomes strongly differ in all studied species [3,36,37,49], reflecting the unique contributions of each RNA chaperone to the post-transcriptional regulation of their host cell. But some degeneracy is also obvious as 10–20% of RNAs can be bound by either protein. Moreover, results of genome-wide studies in *E. coli* [37], *S. enterica* [36] and *N. meningitidis* [49] show that the overlap between the two interactomes is larger than what could be expected by chance (hypergeometric test, *P*<10^−20^), suggesting that co-binding by Hfq and ProQ may be selected for and intentionally exploited by the cell as a regulatory strategy. At the physiological level, both proteins are known to contribute, in non-redundant ways, to the same pathways such as virulence, motility and osmoregulation [37,39,45,49,97].

Focused studies described several scenarios of ProQ-Hfq interplay. The two proteins may engage in direct competition as their binding to the terminator is mutually exclusive [24,60]. In many cases, the effects of each RNA chaperone on their targets are not equivalent. For example, the RaiZ sRNA interacts with both Hfq and ProQ, but only the latter can stabilise RaiZ and mediate its interaction with the *hupA* mRNA [40] and other targets [37]. In a few cases, Hfq and ProQ regulate their shared RNAs in opposite ways. For instance, the AzuCR RNA is destabilised by Hfq but stabilised by ProQ, which also seems to contribute more to the AzuCR-mediated repression of *cadA* and *galE* mRNAs [44]. The RbsZ sponge inactivates the RybB sRNA in an Hfq-dependent mechanism involving RNase III-mediated degradation, but if the same duplex is bound by ProQ, degradation does not occur [37]. These are just a few examples of the regulatory diversity and complexity brought about by combinatorial control through these two RNA chaperones.

## FinO/ProQ-like proteins as evolutionary factors of their hosts

The unique biological properties of the FinO/ProQ family suggest that they are not merely passive bystanders shaped by natural selection but themselves have a huge potential to drive the evolution of their hosts. Several negative and positive mechanisms by which these proteins can affect the tempo and the evolutionary trajectories of bacteria can be contemplated (Figure 6).

We already discussed their multifaceted role of ‘genome stewards’, whereby FinO/ProQ-like proteins repress the transmission and/or expression of selfish genetic elements (plasmids, phages, transposons, toxin–antitoxin systems), prevent acquisition of foreign DNA, and control genes involved in genomic DNA compaction, protection and repair [3,33,34,39–41,49]. Such a role likely stabilises the host genome, slows down its evolution, and in a long run may significantly limit adaptability. Indeed, *proQ* is recurrently mutated or inactivated in adaptive laboratory evolution experiments performed under a variety of conditions [98–102]. At least some *proQ*^-^ lineages were found to acquire mutations much faster, approaching the level of classical mutator strains, which likely accelerates adaptation [103]. From this point of view, FinO/ProQ-family proteins can be regarded as ‘anti-evolution’, conservatory factors.

However, the contribution of FinO- and RocC-like proteins to genome evolution and diversification is likely more nuanced. By repressing their own plasmids, such factors as FinO, FopA and PcnR actually guarantee their survival in the bacterial population. Indeed, conjugative plasmids often show an epidemic burst phase, where they rapidly spread in the population, followed by a ‘dormancy’ phase, where they are simply maintained inside their hosts with as little fitness cost as possible [19,104]. This latter phase depends on the FinO-like proteins. Similarly, what the RocC system offers to *Legionella* is a limited window of competence during which some genome diversification becomes possible. But it also permits to shut down DNA uptake and thereby avoid jeopardising the genome stability [33].

Such ambiguity of contributions seems to exist with respect to other chromosome-encoded ProQ-like proteins as well. It has been recently demonstrated that *proQ* deletion in *S. enterica* shows striking antagonistic pleiotropy [105]. When grown under standard laboratory conditions (LB medium, 37°C), Δ*proQ* strains are actually fitter than their WT parents, in part because they do not waste that much resources on the costly production of flagella [39] and easily outgrow their fellow normal bacteria [105]. Δ*proQ Salmonella* are also uniquely proficient in growth on succinate as the only carbon source, as compared with WT [43]. However, these sudden advantages turn into a handicap in more challenging environments, e.g., during infection or when the same bacteria are exposed to antibiotics. Here, the absence of ProQ dramatically impairs the ability of *Salmonella* to form persister cells and thrive inside the host [39,105]. Together with the above-mentioned plasmid control, which plays an important role in the spread of antibiotic resistances and virulence determinants, these FinO/ProQ-dependent processes must significantly contribute to bacterial adaptation and pathogenicity.

Another potential evolutionary mechanism of broader significance may be provided by the RNA chaperone function of FinO/ProQ-like proteins. It has been observed earlier that the overexpression of RNA chaperones can allow for a higher tolerance of bacteria to disruptive mutations in RNAs (just like high levels of protein chaperones permit to better tolerate destabilising mutations in polypeptides). This mechanism was proposed to be general for RNA chaperones [106]. Interestingly, point mutations in apical loops of FinP usually destroy its pairing with the *traJ* mRNA (which depends on an initial ‘kissing-loop’ interaction). However, the presence of FinO often rescues both the base pairing and regulation [28,29]. This observation suggests that FinO/ProQ-mediated riboregulation might be more tolerant to point mutations in the interacting regions. Such a ‘sanctuary’ role could permit the accumulation of genetic changes in RNA elements without necessarily provoking a loss of function.

But the evolutionary roles of FinO/ProQ-like proteins are likely much more creative than that. What they can offer to cellular transcripts is a defined molecular function (e.g. protection from RNases or base pairing with a target), which is in principle available to any RNA that satisfies their relatively relaxed binding requirements. In other words, they create functional niches for new transcripts. And such novel RNA species do arise. Some come from foreign genetic elements and get integrated into their interactomes: consider the already mentioned RepX sRNA, which imitates FinP to interact with FinO from a different plasmid in *S. enterica* [72], or the plasmid-encoded RocRp sRNA, which mimics the chromosomal RocR sRNA to access RocC and constitutively repress competence in *L. pneumophila* [73]. The globally acting ProQ protein attracted particularly many ‘foreigners’ into its regulon, including sRNAs and mRNAs from genomic islands, toxin–antitoxin loci, transposons and prophages [3].

In a more radical scenario, new functional RNA species can be captured from pervasive transcription or other unorthodox reservoirs if they find a means to be ‘adopted’ by the RBP [107,108]. A major source of evolutionary innovation is exaptation [109]. Indeed, a large number of ProQ-dependent sRNAs arose from 3′-UTRs or CDSs of mRNAs and acquired new, independent biological functions [3,36,37,39,40,62]. Therefore, the presence of ProQ-like proteins in a genetic system may create outstanding evolutionary opportunities to shape transcriptomes and drive innovation.

## Perspectives

The FinO/ProQ family of bacterial RNA chaperones has now been studied quite well from the structural, biochemical, and regulatory points of view across multiple genetic contexts. In contrast, research in their evolutionary aspects, however rich and exciting, is lagging, which does not permit to fully appreciate the genuine scope of this protein family and its unique contribution to biology. Important future directions in this area will include the detailed analysis of FinO/ProQ origins and existing lineages to trace their evolutionary spread and ‘domestication’ events. Furthermore, we need to better understand the evolution of their RNA ligands, in particular sRNAs, and, if possible, correlate it with the evolution of their targets and cognate FinO/ProQ-proteins. An outstanding and still open challenge, despite a number of ground-breaking biochemical and structural studies, is understanding how, and through what evolutionary steps, FinO/ProQ-like proteins could transit between highly specific RBPs (FinO) and globally acting RNA-binding hubs (ProQ). To what extent is this evolution contingent on other players present in the genetic system, especially global ones such as Hfq, CsrA, PNPase or RNase E? Finally, do FinO/ProQ-family proteins significantly influence bacterial evolution? Although in the previous section we suggested an answer to this question, the use of advanced phylogenomic association analyses and experimental evolution approaches will certainly be required to crack this enigma.

## Abbreviations

RBP: RNA-binding protein
sRNA: small noncoding RNA
